# Development of nucleic acid lateral flow immunoassay for molecular detection of *Entamoeba moshkovskii* and* Entamoeba dispar* in stool samples

**DOI:** 10.1038/s41598-024-57332-3

**Published:** 2024-03-19

**Authors:** Sunna Vyatra Hutagalung, Pongruj Rattaprasert, Chamras Promptmas, Saengduen Moonsom, Suganya Yongkiettrakul, Kanthinich Thima, Porntip Chavalitshewinkoon-Petmitr

**Affiliations:** 1 Department of Protozoology, Faculty of Tropical Medicine, Mahidol University, Bangkok, 10400 Thailand; 2 Department of Biomedical Engineering, Faculty of Engineering, Mahidol University, Nakhon Pathom, 73170 Thailand; 3 National Center for Genetic Engineering and Biotechnology, Pathumthani, 12120 Thailand

**Keywords:** Biochemistry, Microbiology, Molecular biology

## Abstract

*Entamoeba moshkovskii*, recently known as a possible pathogenic amoeba, and the non-pathogenic *Entamoeba dispar* are morphologically indistinguishable by microscopy. Although PCR was used for differential diagnosis, gel electrophoresis is labor-intensive, time-consuming, and exposed to hazardous elements. In this study, nucleic acid lateral flow immunoassay (NALFIA) was developed to detect *E. moshkovskii* and *E. dispar* by post-PCR amplicon analysis. *E. moshkovskii* primers were labeled with digoxigenin and biotin whereas primers of *E. dispar* were lebeled with FITC and digoxigenin. The gold nanoparticles were labeled with antibodies corresponding to particular labeling. Based on the established assay, NALFIA could detect as low as 975 fg of *E. moshkovskii* target DNA (982 parasites or 196 parasites/microliter), and 487.5 fg of *E. dispar* target DNA (444 parasites or 89 parasites/microliter) without cross-reactivity to other tested intestinal organisms. After testing 91 stool samples, NALFIA was able to detect seven *E. moshkovskii* (87.5% sensitivity and 100% specificity) and eight *E. dispar* samples (66.7% sensitivity and 100% specificity) compared to real-time PCR. Interestingly, it detected three mixed infections as real-time PCR. Therefore, it can be a rapid, safe, and effective method for the detection of the emerging pathogens *E. moshkovskii* and *E. dispar* in stool samples.

## Introduction

Amoebic infection is mostly prevalent in developing countries. In these countries, the prevalence depends largely on cultural habits, age, level of sanitation, crowding, and socioeconomic status. *Entamoeba histolytica* is the causative agent of invasive amoebiasis; intestinal and extra-intestinal. The other two closely related species; *Entamoeba dispar* and *Entamoeba moshkovskii*, may contribute to confusion in the diagnosis and treatment of amoebiasis, mostly because three of these human *Entamoeba* are morphologically similar. World Health Organization reported that among 500 million people infected with *Entamoeba histolytica*, only 50 million showed symptoms causing mortality in 100,000 deaths^1^. Approximately 80–90% of the infections are asymptomatic and likely due to *Entamoeba dispar*^1^. In developed countries, the infection is mostly due to *E. dispar* and is mostly detained to certain groups: immigrants from or travelers to endemic areas, homosexual males, patients infected with human immunodeficiency virus, and institutionalized populations^2,3^.

While *E. histolytica* is distinctly classified as pathogenic*, E. dispar* is considered to be commensal and non-pathogenic*.* The other closely related species; *E. moshkovskii* was originally thought to be a non-pathogenic protozoan parasite and commonly found in human stool samples in the endemic areas with the misdiagnosis of *E. histolytica* due to their identical morphology under microscopy^4,5^. Beside *E. moshkovskii* in human stool samples has been detected in many countries including the United States, Iran, Turkey, Italy, Australia, Bangladesh, India (Pondicherry), Indonesia, Colombia, Malaysia, Tunisia, Tanzania and Brazil, the occurrence of *E. moshkovskii* in pigs with zoonotic potential from eastern India was recently reported^6^. Although being considered non-pathogenic, an association with gastrointestinal symptoms including diarrhea in human and mice has been gradually found in *E. moshkovskii* infections^7–12^. Moreover, *E. moshkovskii* caused subcutaneous abscesses in Indonesia^13^. In 2023, *E. moshkovskii* infection was mostly found among *Entamoeba*-positive samples from Eastern India, and it was significantly associated with diarrheal incidence indicating its possible emerging enteric pathogen in this region^14^. Interestingly, *E. moshkovskii* and *E. dispar* DNA were detected from liver abscess pus of the patients in India based on PCR assay and confirmed by sequencing highlighting their human health concerns^15^. These findings lead us to think about or reconsider the pathogenicity of these previously known non-pathogenic species”.

*E. histolytica*, *E. dispar* and *E. moshkovskii* are morphologically indistinguishable in their cyst and trophozoite forms from each other by light microscopic findings. The results obtained also depended highly on the expertise of the reader. The sensitivity of the microscopic detection for amoebiasis is estimated around up to only 60%, and prone to false positives due to the misidentification of macrophages, polymorphonuclear leukocytes, tissue cells, and other non-pathogenic species of *Entamoeba*^16–18^. To avoid unnecessary treatment of those with non-pathogenic *Entamoeba* species and to improve understanding of the true burden of amoebiasis, the establishment of an accurate detection method to discriminate each is crucial^18–21^. Molecular biology approach through deoxyribonucleic acid, such as species-specific PCR amplification of *E. histolytica*, *E. dispar* and *E. moshkovskii* DNA^22–24^, may provide improved results. The simultaneous sensing of more than one parasite by multiplex PCR is suggested very efficient^23,24^. However, the non-specific amplification and standardization of multiplex PCR method in endemic areas should be considered^25^. In addition, PCR–RFLP assays were also developed, and showed high sensitivity and specificity for the detection and differentiation of these three species using one or two restriction enzymes generating different RFLP patterns^26,27^. Although it is cheap but the disadvantages of this technique are time-consuming, the requirement of high band density (> 20 ng/µl) in agarose gels for visible RFLP patterns, and misdiagnosis of parasite isolate having the mutation at the restriction site of the enzyme^26,27^. Moreover, PCR‐RFLP assay was still facing practical challenges and was unsuitable for high‐throughput screening. To increase the sensitivity of the diagnosis of *E. histolytica, E. dispar*, and *E. moshkovskii,* multiplex real-time PCR was also developed^28^. However, this multiplex real-time PCR may not be widely used because of the high cost of both instruments and reagents especially in endemic areas. Therefore, PCR assay is more convenient and affordable than real-time PCR in some laboratory settings, especially in developing countries. Since, the examination of amplified PCR products from both the standard PCR assay and the PCR–RFLP still requires the agarose gel electrophoresis, which is labor-intensive, time-consuming, and exposes users to hazardous elements such as ethidium bromide and ultraviolet light. In addition, NALFIA strip can be kept at room temperature so it is portable and convenient for storage and transportation. NALFIA may act as an alternative method for those who are using PCR assay for *E. moshkovskii* and *E. dispar* in their laboratory settings. Therefore, the more rapid and safe method for detection of PCR products such as NALFIA was developed and evaluated in this study.

One of the development areas is paper-based lateral flow assay biosensors^29,30^, including paper-based lateral flow biosensors targeting nucleic acid^30–37^. The application of lateral flow devices as alternative tools for detecting PCR amplicons has been increasingly popular. The development of nanoparticle lateral flow paper-based biosensors is driven by the promise of reaching the best trade-off between rapid performance, affordability, and simplicity^29,33^. Numerous gold nanoparticle-based assays have been developed for biosensor sensing, with label, protein, or DNA-functionalized gold nanoparticles used as the target-specific ligands/probes^30–40^. These include a combination of gold nanoparticles functionalized with labels, proteins, and nucleic acid to assemble sensitive lateral flow assay devices in a sandwich assemblage, with some of them being paper-based.

NALFIA was used to detect hapten-labeled DNA using capture and labeled reporter antibodies or streptavidin. In 2000, NALFIA was first reported for the detection of an opportunistic protozoan infection, *Cryptosporidium parvum* using latex microparticles^41^. After that, the detection of bacteria, *Staphylococcus aureus* using this approach with gold nanoparticles was performed^42^. Since then many NALFIAs have been developed using different hapten labels such as carboxyfluorescein (FAM), digoxygenin (DIG), fluorescein isothiocyanate (FITC), and biotin including SARS-Co-V-2 and influenza detection recently reported^43^. In protozoa, very few reports were found on the use of NALFIA for diagnosis. NALFIAs were developed for the detection of *Plasmodium falciparum* and they could detect low parasite densities^44,45^. In 2017, the detection of *Entamoeba histolytica* using NALFIA was reported but unfortunately, it could not differentiate the other two human *Entamoeba* having similar morphology to *E. histolytica* such as *E. dispar* and *E. moshkovskii*^46^.

Therefore, this study aimed to develop the colorimetric paper-based functionalized gold NALFIA, using anti-labels/antibodies as ligands, for detecting the related labeled target nucleic acid of *E. moshkovskii* and *E. dispar* amplified PCR products. The developed NALFIA can be used as an alternative, rapid, safe, and effective method to the normal PCR process requiring agarose gel electrophoresis and UV transilluminator for visualization of the results.

## Results

### Limit of detection of NALFIA strip for *E. moshkovskii*

**Figure 1:**
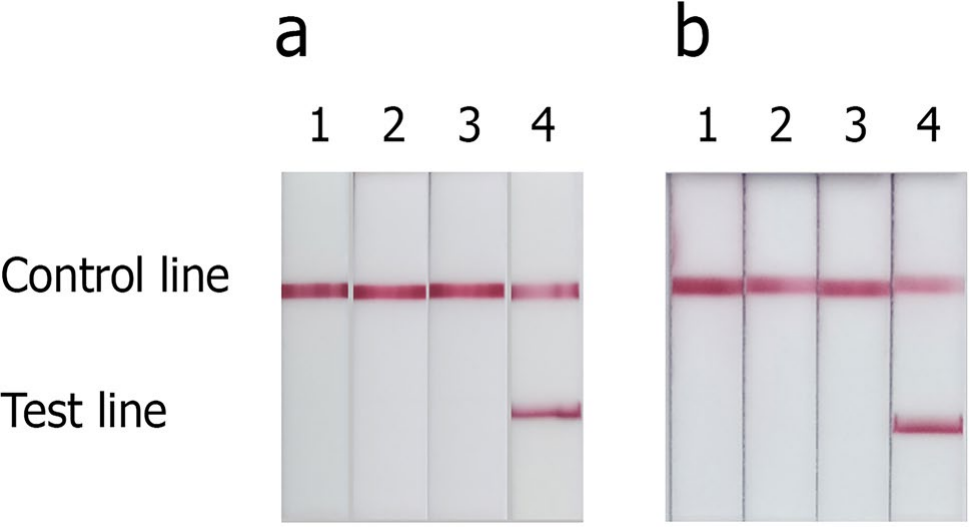
Detection of amplification products of *E. moshkovskii* and *E. dispar* by NALFIA strips. All PCR reactions were performed using species-specific primers as described in the materials and methods. The negative control is the PCR reaction using a particular DNA template without its primers. NALFIA test for *E. moshkovskii* (**a**): strip 1, *E. moshkovskii* negative control; strip 2, *E. histolytica* genomic DNA; strip 3, *E. dispar* plasmid DNA; strip 4, *E. moshkovskii* plasmid DNA. NALFIA test for *E. dispar* (**b**): strip 1, *E. dispar* negative control; strip 2, *E. moshkovskii* plasmid DNA; strip 3, *E. histolytica* genomic DNA; strip 4, *E. dispar* plasmid DNA.

**Figure 2:**
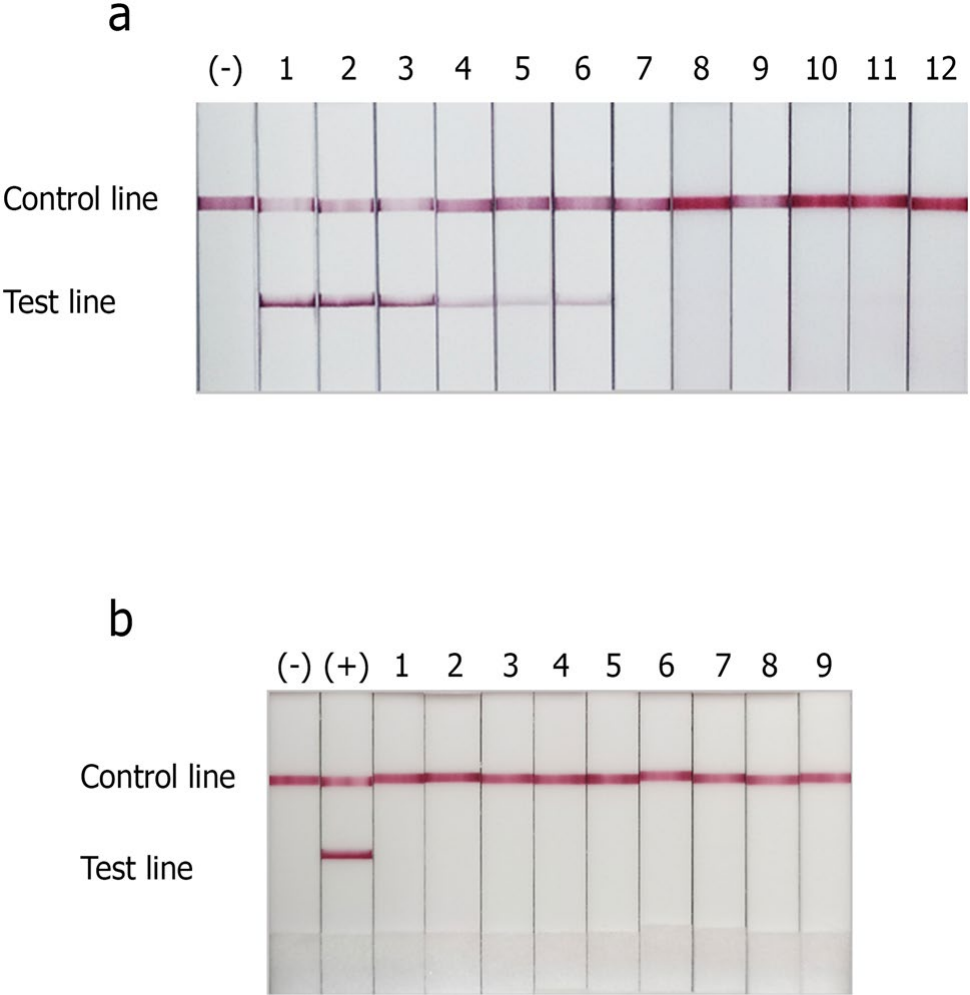
The detection limit and specificity evaluation of *E. moshkovskii* NALFIA. The limit of detection of NALFIA strip was performed using twofold dilutions of *E. moshkovskii* target DNA (**a**). Strip 1, 31.25 pg DNA; strip 2, 15.62 pg DNA; strip 3, 7.81 pg DNA; strip 4, 3.9 pg DNA; strip 5, 1.95 pg DNA; strip 6, 975 fg DNA; strip 7, 487.5 fg DNA; strip 8, 243.75 fg DNA; strip 9, 121.88 fg DNA; strip 10, 60.94 fg DNA; strip 11, 30.47 fg DNA; strip 12, 15.24 fg DNA. Healthy human stool DNA was used as negative control (−)**.** The specificity of *E. moshkovskii* NALFIA was evaluated using amplified products obtained from other known intestinal pathogens (**b**). 1, *G. lamblia* DNA; 2, *Cryptosporidium* *parvum* DNA; 3, *Enterobacter* spp*.* DNA; 4, *K. pneumonia* DNA; 5, *E. coli* bacteria DNA; 6, *Salmonella* group B DNA; 7, *P. mirabilis* DNA; 8, *S. flexneri* DNA; 9, *Acinetobacter* spp*.* DNA. Healthy human stool DNA was used as the negative control (−), and *E. moshkovskii* plasmid DNA was used as the positive control (+).

In this study, NALFIA for the detection of *E. moshkovskii,* and *E. dispar*, was successfully developed. Each type of strip could specifically detect the PCR product of a particular *Entamoeba* species compared with the negative and positive control as shown in Fig. 1. The NALFIA demonstrated the ability to detect *E. moshkovskii* target DNA for as minimum as 975 fg (Fig. 2). Based on calculation for the copy number, *E. moshkovskii* NALFIA could detect 1.97 × 10^5^ copies of plasmid DNA per assay. Since, ribosomal RNA gene in *Entamoeba* is present in high copy number (about 200 copies) per parasite^47,48^, therefore the *E. moshkovskii* NALFIA strip could detect approximately 982 parasites per assay or 196 parasites/µl of DNA template because 5 µl of extracted DNA was used in this PCR reaction. The *E. moshkovskii* NALFIA demonstrated no cross-reactivity with any of these nine pathogenic species, or the DNA from healthy human stool/negative control (Fig. 2).

### Limit of detection of NALFIA strip for *E. dispar*

**Figure 3:**
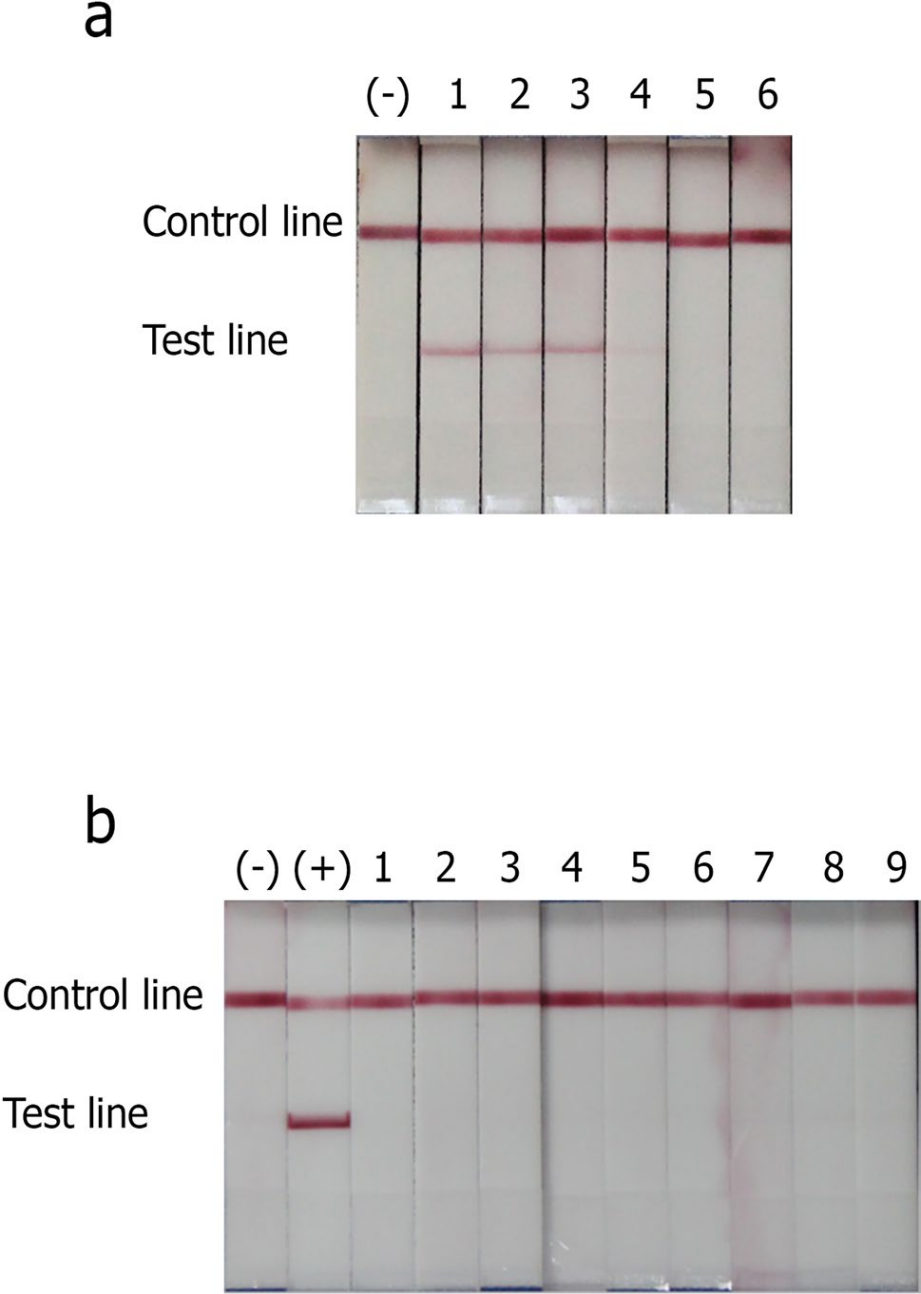
The detection limit and specificity evaluation of *E. dispar* NALFIA. The NALFIA strip was performed using various concentrations of *E. dispar* target DNA, serially diluted into twofold dilutions (**a**). Strip1, 3.9 pg DNA; strip 2, 1.95 pg DNA; strip 3, 975 fg DNA; strip 4, 487.5 fg DNA; strip 5, 243.75 fg DNA; strip 6, 121.88 fg DNA. Healthy human stool DNA was used as negative control (−)**.** The specificity of *E. dispar* NALFIA was tested with amplified products using DNA templates from the other known intestinal pathogens (**b**). 1, *G. lamblia* DNA; 2, *Cryptosporidium* *parvum.* DNA; 3, *Enterobacter* spp*.* DNA; 4, *K. pneumonia* DNA; 5, *E. coli* bacteria DNA; 6, *Salmonella* group B DNA; 7, *P. mirabilis* DNA; 8, *S. flexneri* DNA; 9, *Acinetobacter* spp*.* DNA. Healthy human stool DNA was used as the negative control (−), and *E. dispar* target DNA was used as the positive control (+).

The evaluation of the limit of detection of NALFIA for target DNA of *E.dispar* was as low as 487.5 fg, as shown in Fig. 3. After calculation of copy number, the limit of detection for *E. dispar* plasmid DNA was around 8.88 × 10^4^ copies per assay. Results indicated that *E. dispar* strip was able to detect as low as 444 parasites per assay or at the sample concentration of 89 parasites/µl. The specificity test of *E. dispar* NALFIA strip test was assessed using the known genomic DNAs from the nine species compared with the negative and positive controls. The results demonstrated no cross-reactivity with any of these nine pathogenic species, or the DNA from healthy human stool/negative control (Fig. 3).

### Detection performance of microscopic examination

Based on microscopic examination, both single and mixed infection of protozoan parasites were identified in the positive samples. Of these 91 stool samples, 18 samples (19.8%) were suspected to contain *E. histolytica* according to the characteristics of the parasite cysts whereas 20 samples (21.9%) were positive for *E. histolytica/E. dispar/E. moshkovskii* by the reference method, real-time PCR (Table 1). Moreover, microscopic examination showed five false positive and seven false negative results. Approximately 61.1% (11/18) of these suspected *E. histolytica* positive samples were *E. dispar*, whereas 27.8% (5/18) were identified as *E. moshkovskii* by real-time PCR (Supplementary Table).

**Table 1:** Detection performance of microscopic examination as compared to real-time PCR for *E. histolytica/E. dispar/ E. moshkovskii* on the total 91 stool samples.

		Real-time PCR	
		Positive	Negative
Microscopy* *(E. histolytica/E. dispar/E. moshkovskii)*	Positive	13	5
	Negative	7	66

*The differential diagnosis could not be made by microscopy.

### Detection performance of conventional PCR

According to PCR assay with gel electrophoresis analysis, 88 out of 91 stool samples (96.7%) were negative for both *E. dispar,* and *E. moshkovskii.* PCR results showed 3.3% (3/91) of tested stool samples were positive for *E. dispar* (2 samples) and *E. moshkovskii* (one sample) (Table 2). After further examination, only one *E. histolytica*-positive sample was identified by PCR. Compared with real-time PCR, the conventional PCR exhibited low sensitivity levels as 16.7% and 12.5% for *E. dispar* and *E. moshkovskii* (Table 3). Moreover, the values of NPV, Kappa, and accuracy for diagnosis of both parasites were lower than the NALFIA method.

**Table 2:** Detection performance of PCR and the NALFIA strips as compared with real-time PCR for *E. moshkovskii* and *E. dispar* on the total 91 stool samples.

		Real-time PCR	
		Positive	Negative
PCR			
* E. moshkovskii*	Positive	1	0
	Negative	7	83
* E. dispar*	Positive	2	0
	Negative	10	79
NALFIA			
* E. moshkovskii*	Positive	7	0
	Negative	1	83
* E. dispar*	Positive	8	0
	Negative	4	79

**Table 3:** Performance analysis of PCR and the NALFIA strips as compared to real-time PCR for *E. moshkovskii* and *E. dispar* detection in stool samples.

	Cohen’s kappa compared to real-time PCR*	Sensitivity (%)	Specificity (%)	PPV (%)	NPV (%)	Accuracy (%)
PCR *E. moshkovskii*	0.207	12.5	100	100	92.2	92.3
PCR *E. dispar*	0.258	16.67	100	100	88.8	89
NALFIA *E. moshkovskii*	0.927	87.5	100	100	98.8	98.9
NALFIA *E. dispar*	0.776	66.7	100	100	95.2	95.6

*The Cohen’s kappa coefficient was interpreted as:

0, No agreement;

0.01–0.20, slight agreement;

0.21–0.40, fair agreement;

0.41–0.60, moderate agreement;

0.61–0.80, substantial agreement;

0.81–1.00, almost perfect agreement.

### Detection performance of real-time PCR

After testing all 91 samples by real-time PCR, twelve were positive for *E. dispar* and eight were positive for *E. moshkovskii* (Table 2). Among these positive samples, three of them were mixed infections of *E. dispar* and *E. moshkovskii* and one sample was mixed infection with *E. histolytica*. In addition, 4.4% (4/91) were positive for *E. histolytica* by real-time PCR, showing a four times higher number of positive cases than PCR. Moreover, 37.5% (3/8) of *E. moshkovskii* positive samples detected by real-time PCR were reported as non-pathogenic *Entamoeba coli*; a species of the same genus; *Entamoeba* spp by microscopy.

### Detection performance of the NALFIA strip

**Figure 4:**
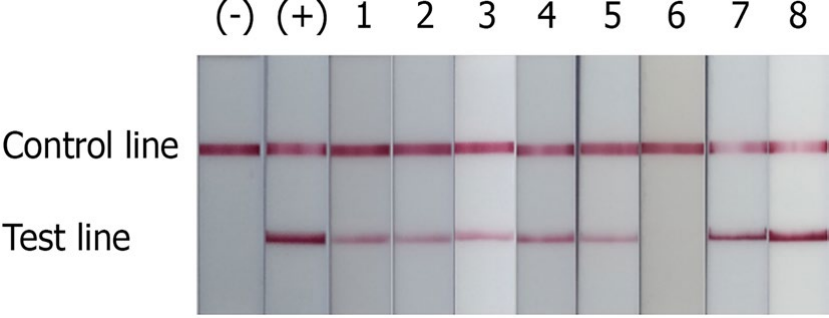
Performance of the NALFIA strips for detection of *E. moshkovskii* in eight positive stool samples detected by real-time PCR. Healthy human stool DNA was used as a negative control (−), and *E. moshkovskii* target DNA was used as a positive control (+).

**Figure 5:**
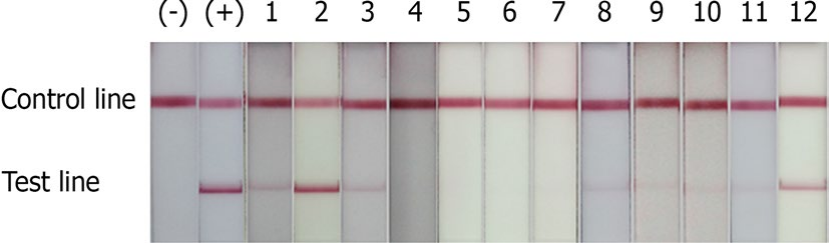
Performance of the NALFIA strips detecting *E. dispar* in 12 positive stool samples reported by real-time PCR. Healthy human stool DNA was used as a negative control (-), and *E. dispar* plasmid DNA was as a positive control (+).

The evaluation of NALFIA strip test for specific detection of *E. dispar* and *E. moshkovskii* was performed and results were compared with PCR and real-time PCR (Table 2). By using the *E. dispar* NALFIA strip test to detect the PCR product, eight out of twelve samples were positive, showing signals on TL (Fig. 4). From prior aforementioned results, eight samples were proven to contain *E. moshkovskii* by real-time PCR, including five samples of single infection, and three cases of mixed infection with *E. dispar* and *E. moshkovskii*. Of these positive cases by real-time PCR, only one sample showed a PCR product band of 580 bp, related to *E. moshkovskii*, by conventional PCR whereas the *E. moshkovskii* NALFIA strip test was able to detect seven positive samples (Table 2) with demonstrated TL signals (Fig. 5). Interestingly, the NALFIA demonstrated three samples of *E. dispar* and *E. moshkovskii* mixed infection as shown by real-time PCR.

A summary of the NALFIA performance is demonstrated in Table 3. The NALFIA successfully detected the presence of *E. dispar* in stool samples, with a sensitivity of 66.67% and specificity of 100%, as compared to real-time PCR. The NALFIA also correctly detected the presence of *E. moshkovskii* in clinical samples, with a sensitivity of 87.5% and specificity of 100%, as compared to real-time PCR. There was no cross-reactivity or false positive found in all the NALFIA-tested clinical samples (PPV = 100%). The NALFIA strips of *E. moshkovskii* showed a significant result that was in almost perfect agreement when compared with a reference standard, real-time PCR (Kappa = 0.927). While NALFIA strips of *E. dispar* gave substantial agreement when compared with real-time PCR (Kappa = 0.776).

## Discussion

Microscopy is widely available and is an affordable method to examine *E. histolytica* suspected clinical samples. However, this method cannot differentiate *E. histolytica* from the other two species; *E. dispar* and *E. moshkovskii*, for a definitive diagnosis. The results obtained also depended highly on the expertise of the microscopist. The sensitivity of the microscopic detection in this study was estimated to be up to only 60%. This was as expected, due to the experience and expertise from the microscopic readers. Nevertheless, there were also findings of false positive and false negative cases. Similar findings were also reported from other previous studies^16–18^. In most cases, for screening purpose, a method with a high degree of sensitivity and lower specificity is recommended, whereas for diagnosis purpose, a method with a high degree of both sensitivity and specificity is required^49^. Therefore, it is advisable to apply microscopic examination for screening purposes, followed by more specific examination methods for definitive diagnosis.

Antigen detection is generally considered superior to microscopy. Though it was previously described as comparable performance to PCR^50,51^, further findings revealed dissatisfactory performance reports^52,53^. Antigen detection faces challenges from cross-reactivity and the application of sample fixation because it requires a large amount of trophozoites. Currently, no specific antibody/antigen tests are currently commercially available for the detection of *E. dispar* and *E. moshkovskii* from clinical samples.

Molecular biology methods, such as species-specific PCR amplification of *E. histolytica*, *E. dispar,* and *E. moshkovskii* DNA can provide high sensitivity and specificity for diagnosis and differentiation of these target species^23,54–56^. Despite this superiority, it requires post-PCR analysis by agarose gel electrophoresis, which is labor-intensive, time-consuming, and may expose users to hazardous elements such as ethidium bromide and ultraviolet light. More studies offered the idea of replacing agarose gel electrophoresis with the paper-based lateral flow biosensor, targeting the nucleic acid, for post-PCR amplicons analysis. There are numerous reported NALFIA developments by application of labels on the primers used^30,37,57^. Additionally, there are also published studies employing the labels on the probe/s instead of only labels on the primers^58–60^. Nevertheless, the application of probe/s requires additional step in the analysis process, and hybridization of probe/s with the target product and conjugates necessitates further optimization. This study applied the labels on the primers used. It offered the combination of PCR species-specific amplification power, the gold nanoparticle’s unique properties as the transducer, the selectivity of the applied labels as the biorecognition molecules, and the high efficiency of chromatographic separation, for target *E. histolytica/E. dispar/E. moshkovskii* post-PCR analysis.

In this study, the NALFIA as the alternative post-PCR analysis for detection of *E. moshkovskii* and *E. dispar* was successfully developed. Apart from our primers used in the PCR assay, the detection limit of *E. moshkovskii* and *E. dispar* using different primers, techniques, and settings was available from a few studies compared with *E. histolytica*. The sensitivity of PCR–RFLP showed the lowest detection limit as 2.6 pg genomic DNA of both species^27^. The limit of detection of the tetraplex PCR was found to be 78 pg genomic DNA or 1000 parasites of *E. dispar*^61^ which was higher than the current method using NALFIA (444 parasites per assay). The detection limit of nested multiplex PCR for *E. dispar* and *E. moshkovskii* was approximately 25 parasites per assay^23^ which was lower than the current method. However, results of the NALFIA test can be obtained within 2 h of receipt of stool samples compared with 12 h from the nested multiplex PCR. The current assay using combination of PCR and NALFIA strip is rapid and convenient in detecting *E. moshkovskii* and *E. dispar*, even when the adjusted primer concentration used in the assay is lower than the concentration used in our previous study^24^.

For evaluation against clinical specimens for targeting *E. dispar*, although the assay accuracy only offered a sensitivity of 66.67% as compared to real-time PCR result, the performance was significantly better than gel electrophoresis which only offered a sensitivity of 16.67% (Table 3). The developed assay also has good specificity with no cross-reactivity found. For evaluation against clinical specimen for targeting *E. moshkovskii*, the assay accuracy offered an excellent sensitivity of 87.5% as compared to real-time PCR results, and the performance was significantly better than gel electrophoresis which only offered a sensitivity of 12.5% (Table 3), and there was no cross-reaction found in the specificity test. The current results showed that the developed NALFIA provided increased sensitivity for the diagnosis of *E. dispar* and *E. moshkovskii*. A previous study on lateral flow also showed similar results, expressing that the performance of lateral flow is better than gel electrophoresis for post-PCR analysis^62^. The cost of PCR and NALFIA strip for detection of *E. moshkovskii* and *E. dispar* is about 15 dollars per test which two dollars are for NALFIA strip. The inexpensive cost of using NALFIA and other advantages previously mentioned compared with gel electrophoresis for post-PCR detection indicate its cost-effectiveness especially for the examination of a large number of tested stool samples.

According to our results, the distribution pattern of each target species in stool samples; dominated by *E. dispar*, was similar to the findings from other previous studies^10,63,64^. Interestingly, the higher number of *E. moshkovskii*-positive field samples in Thailand was found in this study compared with the previous report showing only one case was found for the presence of *E. moshkovskii*^28^. Although the tested samples were adequate for statistical analysis, further investigation needs to be carried out with the larger sample size in wider geographical areas to obtain better evaluation of specificity and sensitivity of this combining PCR with the NALFIA.

To our knowledge, this is the first lateral flow assay for post-PCR analysis targeting *E. dispar* and *E. moshkovskii*. As an emerging pathogen, *E. moshkovskii* is associated with gastrointestinal symptoms, including diarrhea^7–10^. Hence, the availability of improved methods for detecting *E. moshkovskii* is also crucial. This study found excellent sensitivity and specificity of the developed NALFIA for targeting *E. moshkovskii*. The NALFIA as an alternative for post-PCR analysis, requires a shorter time to complete the process. It is portable in dry form and does not require extra instruments such as a gel casting tray and combs, an electrophoresis chamber and power supply, and an ultraviolet transilluminator for gel visualization. Combining NALFIA with nanoparticles as the transducer; such as a gold nanoparticle, also offers the possibility for increased sensitivity. In some settings where real-time PCR facility is not available, this NALFIA assay for post-PCR analysis targeting *E. moshkovskii* and *E. dispar* can be used as a platform that can offer a rapid, safer, and more convenient method as compared to gel electrophoresis analysis for these two *Entamoeba* causing health problems worldwide.

## Materials and methods

### DNA samples

The positive control DNA of *E. moshkovskii* and *E. dispar* was obtained from the cloning of the SSU rRNA genes for *E. moshkovskii* and *E. dispar* into plasmid synthesized by Macrogen, Inc., South Korea, then the plasmids were used as reference. *E. histolytica* DNA was extracted from axenically grown *E. histolytica* (HM-1: IMSS)^65^ and used as a positive control.

### Stool samples

In total, 91 stored stool samples were used to evaluate the NALFIA performance. These human stool samples were obtained from different areas in Thailand during the routine community service provided by the Department of Protozoology, Faculty of Tropical Medicine, Mahidol University. All stool samples were screened and confirmed for protozoan infection through microscopy. Then DNA was extracted by using the PSP® Spin Stool DNA Kit (STRATEC Molecular GmbH, D-13125 Berlin, Germany), and stored at − 80 °C until further use. Extracted DNA of stored samples kept from 2015 to 2020 were used in this study.

### PCR amplification

The sequences of forward primer (FP) and reverse primer (RP) used were based on our previous work^24^, with slight modifications to the amplification parameters. The PCR amplification reaction used Taq DNA polymerase with a standard Taq buffer kit (New England BioLabs, USA). The reaction with a final volume of 50 µl was performed by Eppendorf Master Cycler Pro S (Eppendorf AG, Germany). The reaction mixture contained 200 µM of each deoxynucleoside triphosphate, 0.2 µM of each genus-specific forward primer (EntaF) and species-specific reverse primers (EhR or EdR or EmR), 1.25 U of Taq polymerase, 1.5 mM MgCl_2_, 1× Taq buffer, and 5 µl of extracted DNA, amplified in single-round PCR for each target species. For positive control reactions, 2.5 ng of genomic DNA of *E. histolytica* genomic DNA and 1 ng of plasmid DNA containing small subunit rRNA (18S rRNA) gene sequences of *E. moshkovskii* and *E. dispar* were used as reference DNA templates.

The amplification started with an initial denaturation at 95 °C for 3 min, followed by 40 cycles of 95 °C for 30 s, 55 °C for 30 s, and 68 °C for 30 s, with a final extension at 68 °C for 3 min. The amplified products were analyzed using 1.5% agarose gels, followed by ethidium bromide staining, and subsequently visualized under UV light (Supplementary Fig. 1).

### Development of the NALFIA

#### Design of primers and labels for the assay

**Figure 6:**
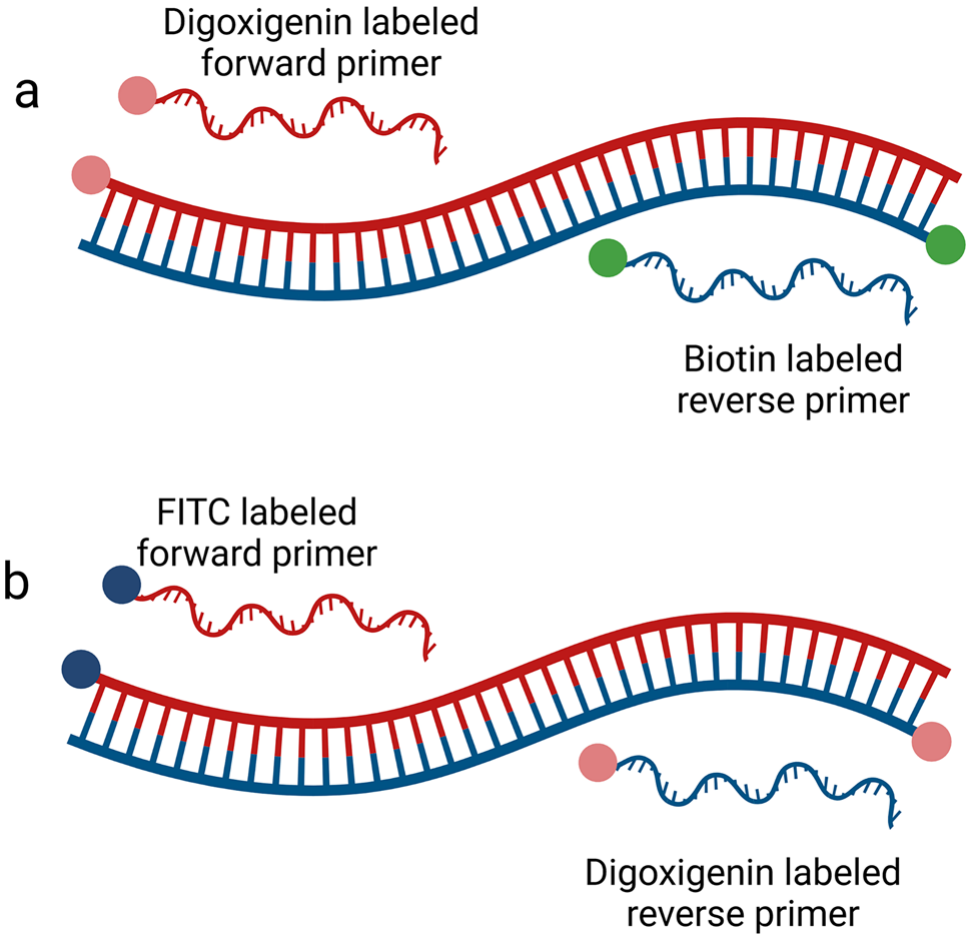
The PCR amplification concept for each of the target species. The FP and RP are labeled accordingly: *E. moshkovskii* (**a**), and *E. dispar* (**b**)*.*

The sequences of primers used were similar to the ones used for the PCR amplification with gel electrophoresis analysis^24^. The assigned label for each primer is demonstrated in Fig. 6. In this scheme, there are two pair sets of labels applied for each target species.

In the assay targeting *E. moshkovskii*, the genus-specific FP (EntaF) was labeled with digoxigenin; then called EntaF-digoxigenin, and the species-specific RP (EmR) was labeled with biotin; then called EmR-biotin (Fig. 6). The forward and reverse primers for *E. moskovskii* were designed as EntaF-digoxigenin: 5′-digoxigenin-ATG CAC GAG AGC GAA AGC AT-3′, and EmR-biotin: 5′-biotin-TGA CCG GAG CCA GAG ACA T-3′. The binding of the PCR amplification product containing sequence related to *E. moshkovskii* to the gold nanoparticle was done by labeling the nanoparticle with the primary anti-digoxigenin antibody. Then the paper was labeled with anti-biotin on the test line (TL) for immobilization.

For targeting *E. dispar*, the genus-specific primer (EntaF) was labeled with FITC and named as EntaF-FITC, and the species-specific RP (EdR) was labeled with digoxigenin; then called EdR-digoxigenin (Fig. 6). The primer sequences and labelings for forward and reward primers of *E. dispar* were EntaF-FITC: 5′-FITC-ATG CAC GAG AGC GAA AGC AT-3′, and EdR-digoxigenin: 5′-digoxigenin-CAC CAC TTA CTA TCC CTA CC-3′. The nanoparticle was labeled with the primary anti-FITC antibody. For the immobilization of the paper, an anti-digoxigenin antibody was added to the TL area. To ensure that the assay would function properly, the anti-species antibody was immobilized further up; at the control line (CL) area of the paper.

### Construction of NALFIA strips

**Figure 7:**
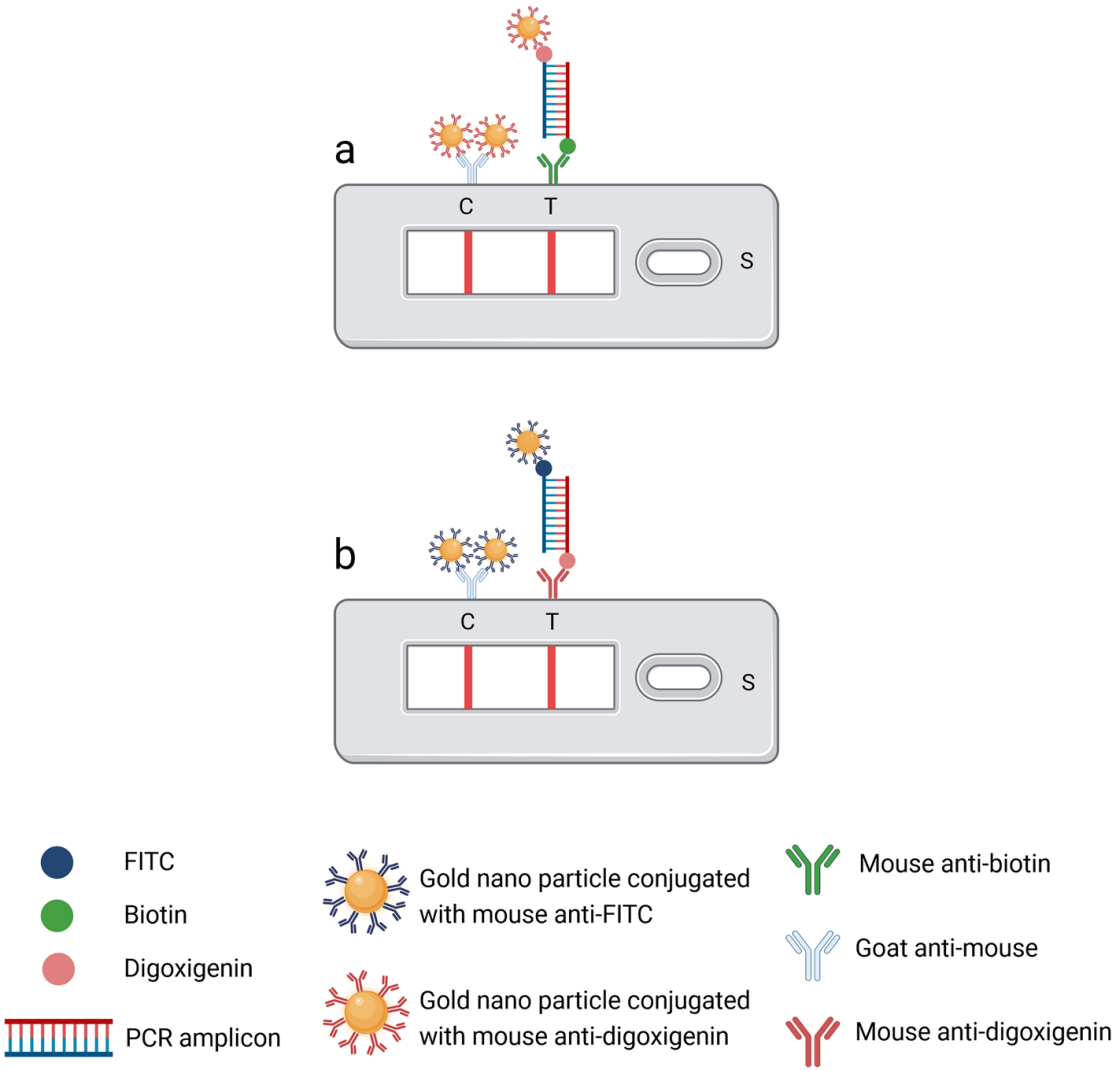
Principle of the assay: *E. moshkovskii* detection (**a**), and *E. dispar* detection (**b**).

All tested strips were fabricated and manufactured by Kestrel BioSciences Thailand Co. Ltd. based on our design. Figure 7 illustrates a schematic principle of the PCR product captured on detection pad of NALFIA strip. In principle, the fabricated strip sized 3 × 80 mm each, consists of four components: sample application pad, conjugate pad, nitrocellulose membrane/detection pad (with TL and CL), and absorbent pad. According to the information from the manufacturer, the conjugate used in the strip was described as a gold nanoparticle of 40 nm diameter, coated with labels and conjugated by the passive adsorption method. For *E. moshkovskii*, the gold nanoparticle was labeled with mouse anti-digoxigenin to create the 10 OD conjugate dispensed onto the conjugate pad by using BioDot XYZ3060™ Dispense System, USA. For *E. moshkovskii* detection, the anti-species antibody (the goat anti-mouse IgG) of 1 mg/ml as CL and the 1 mg/ml mouse anti-biotin as TL were dispensed onto the detection pad (Fig. 7). In *E. dispar*, the gold nanoparticle was labeled with mouse anti-FITC to create the 10 OD conjugate dispensed onto the conjugate pad. For *E. dispar* detection, the anti-species antibody (the goat anti-mouse IgG) of 1 mg/ml for CL and 1 mg/ml of mouse anti-digoxigenin for TL were dispensed onto the detection pad (Fig. 7).

### PCR amplification for the NALFIA test

For the PCR assay, the reaction mixture contained 200 µM of each deoxynucleoside triphosphate, each labeled FP and RP for the target species, 1.25 U of *Taq* polymerase, 1× *Taq* buffer, and 5 µl of extracted DNA. Each FP and RP concentration was 20 nM for detecting *E. moshkovskii* per reaction, and 30 nM for identifying *E. dispar* per reaction. Where applicable, for positive control, the template for reference DNA applied was 1 ng per reaction for *E. moshkovskii* and *E. dispar* DNA. Amplification of each species-specific DNA fragment started with an initial denaturation at 95 °C for 3 min, followed by 40 cycles of 95 °C for 45 s, 55 °C for 45 s, and 68 °C for 45 s, with a final extension at 68 °C for 5 min. The whole amplified products were then directly used for the lateral flow assay.

### Lateral flow assay

In the assay, the whole 50 µl of the PCR product containing target DNA was mixed in 200 µl running buffer (Kestrel Bioscience, Thailand), and the strip was immersed into this solution until the flow reached the absorbent pad level. Subsequently, another 100 μl of the running buffer was dispensed to wash away the excess conjugate solution and then left until the liquid stopped at the top area of the absorbent pad. The assay was completed and observed within 15 min.

### Limit of detection of combining PCR with NALFIA assay

The combining PCR amplification with the lateral flow assay or NALFIA was evaluated for its limit of detection (LOD) of the sample. The known amount of target DNA of each species was serially diluted by twofold dilution of the DNA template for PCR reaction. According to 5 µl of DNA template was used in each PCR, the evaluation was started from preparation the DNA template at the concentration of 31.25 pg of plasmid DNA/5 µl and serially diluted by twofold to the concentration of 15.24 fg/5 µl of *E. moshkovskii* plasmid DNA and used as templates in PCR assay. After amplification products were obtained and mixed with 200 µl running buffer (Kestrel BioSciences, Thailand), the NALFIA strip was immersed into the solution. The positive signal was observed within 15 min. To set the lowest detection limit of the PCR-NALFIA assay for *E. dispar*, the started plasmid DNA at concentration of 3.9 pg/5 µl was twofold serially diluted to the concentration of 121.88 fg/5 µl of plasmid DNA and added to each PCR assay and then PCR product was detected by NALFIA strip for *E. dispar* under the same conditions specified before.

The corresponding copy number of the amount of plasmid DNA at the lowest detectable level was calculated by using the following equation^66,67^.

DNA (copy) = 6.02 × 10^23^(copy/mol) × DNA amount(g)/DNA length(bp) × 660(g/mol/bp).

### Specificity of combining PCR with NALFIA assay

The developed NALFIA was evaluated for its specificity against genomic DNAs of nine commonly found intestinal bacterial or protozoal pathogens; *G. lamblia* (ATCC 50803)*, Cryptosporidium parvum* (Iowa strain)*, Enterobacter* spp*., Klebsiella pneumonia*, *Escherichia coli, Salmonella* group B*, Proteus mirabilis, Shigella flexneri,* and *Acinetobacter* spp. All bacterial DNA were from cultured isolates and provided by the Department of Microbiology and Immunology, Faculty of Tropical Medicine, Mahidol University. The amount of DNA template applied for each target was 2.5 ng, amplified, and tested to check any cross-reactivity. Where applicable, for positive control, the template for reference DNA applied was 2.5 ng per reaction for *E. histolytica* genomic DNA, and 1 ng/reaction for *E. dispar* and *E. moshkovskii* plasmid DNA. Extracted DNA from healthy human stools was used as a negative control.

### Data analysis

The analysis of all obtained data was performed by using Stata® 12.0 (StataCorp, USA) software. Performance of NALFIA as sensitivity and specificity was comparatively analyzed to the reference method (real-time PCR). The positive predictive value (PPV), negative predictive value (NPV), and accuracy were calculated^68^. In addition, Kappa statistics were used to evaluate the diagnostic performance of NALFIA and conventional PCR compared to real-time PCR.

### Ethics approval

This study was reviewed and approved by the Ethics Committee of the Faculty of Tropical Medicine, Mahidol University (certificate of ethical approval number MUTM 2017–009-01). Informed consent requirement was waived by the Ethics Committee of the Faculty of Tropical Medicine, Mahidol University for this study. All experiments and analyses were executed according to the approved guidelines and relevant regulations.

### Supplementary Information

Supplementary Table 1.

Supplementary Legends.

Supplementary Figure 1.

## Data Availability

All generated or analyzed data during this research study are included in this published article and the supplementary file.

## References

[1] Lozano R (2012). Global and regional mortality from 235 causes of death for 20 age groups in 1990 and 2010: A systematic analysis for the Global Burden of Disease Study 2010. Lancet.

[2] Petri WA (1996). Recent advances in amebiasis. Crit. Rev. Clin. Lab. Sci..

[3] Ali IK (2015). Intestinal amebae. Clin. Lab. Med..

[4] Clark CG, Diamond LS (1991). The Laredo strain and other '*Entamoeba histolytica*-like' amoebae are *Entamoeba moshkovskii*. Mol. Biochem. Parasitol..

[5] Clark CG, Diamond LS (1992). Differentiation of pathogenic *Entamoeba histolytica* from other intestinal protozoa by riboprinting. Arch Med. Res..

[6] Sardar, S.K. *et al.* Molecular evidence suggests the occurrence of *Entamoeba moshkovskii* in pigs with zoonotic potential from eastern India. *Folia Parasitol. (Praha)* **69** (2022). 10.14411/fp.2022.012 35727049

[7] Shimokawa C (2012). *Entamoeba moshkovskii* is associated with diarrhea in infants and causes diarrhea and colitis in mice. J. Infect. Dis..

[8] Fotedar R, Stark D, Marriott D, Ellis J, Harkness J (2008). *Entamoeba moshkovskii* infections in Sydney, Australia. Eur. J. Clin. Microbiol. Infect. Dis..

[9] Yakoob J (2012). Entamoeba species associated with chronic diarrhoea in Pakistan. Epidemiol. Infect..

[10] Anuar TS (2012). First molecular identification of *Entamoeba moshkovskii* in Malaysia. Parasitology.

[11] Tanyuksel M (2007). Two cases of rarely recognized infection with *Entamoeba moshkovskii*. Am. J. Trop. Med. Hyg..

[12] Shimokawa C (2018). Intestinal inflammation-mediated clearance of Amebic parasites is dependent on IFN-gamma. J. Immunol..

[13] Bs SH (2018). Comparison of multiplex single round Pcr and microscopy in diagnosis of amoebiasis. Afr. J. Infect. Dis..

[14] Sardar SK (2023). Prevalence and molecular characterization of *Entamoeba moshkovskii* in diarrheal patients from Eastern India. PLoS Negl. Trop. Dis..

[15] Kumar M, Nath G, Parija SC (2016). Detection of *Entamoeba dispar* and *Entamoeba moshkovskii* DNA in liver abscess pus: Newer perspectives to be considered in diagnosis of amoebiasis. Int. J. Infect. Dis..

[16] Pillai DR (1999). *Entamoeba histolytica* and *Entamoeba dispar*: epidemiology and comparison of diagnostic methods in a setting of nonendemicity. Clin. Infect. Dis..

[17] Walsh JA (1986). Problems in recognition and diagnosis of amebiasis: Estimation of the global magnitude of morbidity and mortality. Rev. Infect. Dis..

[18] Tanyuksel M, Petri WA (2003). Laboratory diagnosis of amebiasis. Clin. Microbiol. Rev..

[19] Huston CD, Haque R, Petri WA (1999). Molecular-based diagnosis of *Entamoeba histolytica* infection. Expert. Rev. Mol. Med..

[20] Roy S (2005). Real-time-PCR assay for diagnosis of *Entamoeba histolytica* infection. J. Clin. Microbiol..

[21] Organization, W.H. Amoebiasis. *WHO Weekly Epidemiological Record*, 97–100 (1997).

[22] Ali IK (2003). *Entamoeba moshkovskii* infections in children, Bangladesh. Emerg. Infect. Dis..

[23] Khairnar K, Parija SC (2007). A novel nested multiplex polymerase chain reaction (PCR) assay for differential detection of *Entamoeba histolytica*, *E. moshkovskii* and *E. dispar* DNA in stool samples. BMC Microbiol..

[24] Hamzah Z, Petmitr S, Mungthin M, Leelayoova S, Chavalitshewinkoon-Petmitr P (2006). Differential detection of *Entamoeba histolytica*, *Entamoeba dispar*, and *Entamoeba moshkovskii* by a single-round PCR assay. J. Clin. Microbiol..

[25] Fotedar, R. *et al.* Laboratory diagnostic techniques for *Entamoeba* species. *Clin. Microbiol. Rev.* **20**, 511–532, table of contents (2007). 10.1128/CMR.00004-07 PMC1932757 17630338

[26] Fontecha GA (2015). A PCR-RFLP method for the simultaneous differentiation of three *Entamoeba* species. Exp. Parasitol..

[27] Sardar SK (2023). Development of a simple PCR–RFLP technique for detection and differentiation of *E. histolytica*, *E. dispar* and *E. moshkovskii*. Parasitol. Res..

[28] Hamzah Z, Petmitr S, Mungthin M, Leelayoova S, Chavalitshewinkoon-Petmitr P (2010). Development of multiplex real-time polymerase chain reaction for detection of *Entamoeba histolytica*, *Entamoeba dispar*, and *Entamoeba moshkovskii* in clinical specimens. Am. J. Trop. Med. Hyg..

[29] Ahmed S, Bui MP, Abbas A (2016). Paper-based chemical and biological sensors: Engineering aspects. Biosens. Bioelectron..

[30] Chua A, Yean CY, Ravichandran M, Lim B, Lalitha P (2011). A rapid DNA biosensor for the molecular diagnosis of infectious disease. Biosens. Bioelectron..

[31] Gao X (2014). Visual detection of microRNA with lateral flow nucleic acid biosensor. Biosens. Bioelectron..

[32] He Y (2011). Ultrasensitive nucleic acid biosensor based on enzyme-gold nanoparticle dual label and lateral flow strip biosensor. Biosens. Bioelectron..

[33] Liu CC, Yeung CY, Chen PH, Yeh MK, Hou SY (2013). Salmonella detection using 16S ribosomal DNA/RNA probe-gold nanoparticles and lateral flow immunoassay. Food Chem..

[34] Mao X (2009). Disposable nucleic acid biosensors based on gold nanoparticle probes and lateral flow strip. Anal. Chem..

[35] Mao, X., Xu, H., Zeng, Q., Zeng, L. & Liu, G. Molecular beacon-functionalized gold nanoparticles as probes in dry-reagent strip biosensor for DNA analysis. *Chem. Commun. (Camb.)*, 3065–3067 (2009). 10.1039/b822582f 19462088

[36] Wu W (2015). A sensitive lateral flow biosensor for *Escherichia coli* O157:H7 detection based on aptamer mediated strand displacement amplification. Anal Chim Acta.

[37] Rivas L (2015). Triple lines gold nanoparticle-based lateral flow assay for enhanced and simultaneous detection of *Leishmania* DNA and endogenous control. Nano Res..

[38] Mao X, Du TE, Meng L, Song T (2015). Novel gold nanoparticle trimer reporter probe combined with dry-reagent cotton thread immunoassay device for rapid human ferritin test. Anal. Chim Acta.

[39] Parolo C, de la Escosura-Muniz A, Merkoci A (2013). Enhanced lateral flow immunoassay using gold nanoparticles loaded with enzymes. Biosens. Bioelectron..

[40] Liu G (2009). Aptamer-nanoparticle strip biosensor for sensitive detection of cancer cells. Anal. Chem..

[41] Kozwich D (2000). Development of a novel, rapid integrated *Cryptosporidium parvum* detection assay. Appl. Environ. Microbiol..

[42] Fong WK (2000). Rapid solid-phase immunoassay for detection of methicillin-resistant *Staphylococcus aureus* using cycling probe technology. J. Clin. Microbiol..

[43] Akalin P, Yazgan-Karatas A (2023). Development of a nucleic acid-based lateral flow device as a reliable diagnostic tool for respiratory viral infections. MethodsX.

[44] Mens PF (2012). Direct blood PCR in combination with nucleic acid lateral flow immunoassay for detection of *Plasmodium* species in settings where malaria is endemic. J. Clin. Microbiol..

[45] Roth JM (2018). Plasmodium detection and differentiation by direct-on-blood PCR nucleic acid lateral flow immunoassay: Development, validation, and evaluation. J. Mol. Diagn..

[46] Foo PC (2017). Development of a thermostabilised triplex LAMP assay with dry-reagent four target lateral flow dipstick for detection of *Entamoeba histolytica* and non-pathogenic *Entamoeba* spp. Anal. Chim Acta.

[47] Bhattacharya S, Bhattacharya A, Diamond LS (1988). Comparison of repeated DNA from strains of *Entamoeba histolytica* and other *Entamoeba*. Mol. Biochem. Parasitol..

[48] Chihi, A., O'Brien Andersen, L., Aoun, K., Bouratbine, A. & Stensvold, C.R. Amplicon-based next-generation sequencing of eukaryotic nuclear ribosomal genes (metabarcoding) for the detection of single-celled parasites in human faecal samples. *Parasite Epidemiol. Control* **17**, e00242 (2022). 10.1016/j.parepi.2022.e00242 PMC8819130 35146142

[49] Bujang, M.A. & Adnan, T.H. Requirements for minimum sample size for sensitivity and specificity analysis. *J. Clin. Diagn. Res.* **10**, YE01-YE06 (2016). 10.7860/JCDR/2016/18129.8744 PMC5121784 27891446

[50] Haque R (2000). Diagnosis of amebic liver abscess and intestinal infection with the TechLab *Entamoeba histolytica* II antigen detection and antibody tests. J. Clin. Microbiol..

[51] Haque R, Neville LM, Hahn P, Petri WA (1995). Rapid diagnosis of *Entamoeba* infection by using *Entamoeba* and *Entamoeba histolytica* stool antigen detection kits. J. Clin. Microbiol..

[52] Gonin P, Trudel L (2003). Detection and differentiation of *Entamoeba histolytica* and *Entamoeba dispar* isolates in clinical samples by PCR and enzyme-linked immunosorbent assay. J. Clin. Microbiol..

[53] Zeehaida M (2008). A study on the usefulness of Techlab *Entamoeba histolytica* II antigen detection ELISA in the diagnosis of amoebic liver abscess (ALA) at Hospital Universiti Sains Malaysia (HUSM), Kelantan, Malaysia. Trop. Biomed..

[54] Ngui R (2012). Differentiating *Entamoeba histolytica*, *Entamoeba dispar* and *Entamoeba moshkovskii* using nested polymerase chain reaction (PCR) in rural communities in Malaysia. Parasit. Vectors.

[55] ElBakri A, Samie A, Ezzedine S, Odeh RA (2013). Differential detection of *Entamoeba histolytica*, *Entamoeba dispar* and *Entamoeba moshkovskii* in fecal samples by nested PCR in the United Arab Emirates (UAE). Acta Parasitol..

[56] Fotedar R (2007). PCR detection of *Entamoeba histolytica*, *Entamoeba dispar*, and *Entamoeba moshkovskii* in stool samples from Sydney, Australia. J. Clin. Microbiol..

[57] Horng YT (2006). Development of an improved PCR-ICT hybrid assay for direct detection of *Legionellae* and *Legionella pneumophila* from cooling tower water specimens. Water Res..

[58] Wang J (2012). A novel, universal and sensitive lateral-flow based method for the detection of multiple bacterial contamination in platelet concentrations. Anal. Sci..

[59] Yongkiettrakul S (2014). Application of loop-mediated isothermal amplification assay combined with lateral flow dipstick for detection of *Plasmodium falciparum* and *Plasmodium vivax*. Parasitol. Int..

[60] Xu Y (2014). Fluorescent probe-based lateral flow assay for multiplex nucleic acid detection. Anal Chem..

[61] Foo PC (2012). Development of a thermostabilized, one-step, nested, tetraplex PCR assay for simultaneous identification and differentiation of *Entamoeba* species, *Entamoeba histolytica* and *Entamoeba dispar* from stool samples. J. Med. Microbiol..

[62] Zhang H (2017). Rapid detection of methicillin-resistant *Staphylococcus aureus* in pork using a nucleic acid-based lateral flow immunoassay. Int. J. Food. Microbiol..

[63] Haghighi A (2018). Amoebiasis in Iran: A systematic review and meta-analysis. Epidemiol. Infect..

[64] Bahrami F, Haghighi A, Zamini G, Khademerfan M (2019). Differential detection of *Entamoeba histolytica*, *Entamoeba dispar* and *Entamoeba moshkovskii* in faecal samples using nested multiplex PCR in west of Iran. Epidemiol. Infect..

[65] Khomkhum N, Leetachewa S, Pawestri AR, Moonsom S (2019). Host-antibody inductivity of virulent *Entamoeba histolytica* and non-virulent *Entamoeba moshkovskii* in a mouse model. Parasit. Vectors.

[66] Whelan JA, Russell NB, Whelan MA (2003). A method for the absolute quantification of cDNA using real-time PCR. J. Immunol. Methods.

[67] Lee C, Kim J, Shin SG, Hwang S (2006). Absolute and relative QPCR quantification of plasmid copy number in *Escherichia coli*. J. Biotechnol..

[68] Hess AS (2012). Methods and recommendations for evaluating and reporting a new diagnostic test. Eur. J. Clin. Microbiol. Infect. Dis..

