# Supplementary material for: Development of nucleic acid lateral flow immunoassay for molecular detection of Entamoeba moshkovskii and Entamoeba dispar in stool samples
Source: Sci Rep. 2024 Mar 19;14:6635. doi: 10.1038/s41598-024-57332-3 (PMC10951296; doi:10.1038/s41598-024-57332-3)
Supplement: Supplementary file 1 — Supplementary Table 1. [file 41598_2024_57332_MOESM1_ESM.docx]

**Table 1.** The results of all 25 positive stool samples for *E. histolytica* (*Eh*)/*E. dispar* (*Ed*)/*E. moshkovskii* (*Em*) detected by microscopy, PCR, real-time PCR, and NALFIA.

Results are shown as positive (+), negative (-), and not tested (NT).

| **No** | **Microscopy**  **(*Eh*-like cyst)** | **PCR** | | | **Real-time PCR** | | | **NALFIA** | | |
| --- | --- | --- | --- | --- | --- | --- | --- | --- | --- | --- |
|  |  | *Eh* | *Ed* | *Em* | *Eh* | *Ed* | *Em* | *Eh* | *Ed* | *Em* |
| 1 | **+** | **-** | **-** | **-** | **-** | **-** | **-** | NT | **-** | **-** |
| 2 | **+** | **-** | **-** | **-** | **-** | **-** | **-** | NT | **-** | **-** |
| 3 | **+** | **-** | **-** | **-** | **-** | **-** | **-** | NT | **-** | **-** |
| 4 | **+** | **-** | **-** | **-** | **-** | **-** | **-** | NT | **-** | **-** |
| 5 | **+** | **-** | **-** | **-** | **-** | **-** | **-** | NT | **-** | **-** |
| 6 | **-** | **-** | **-** | **-** | **+** | **-** | **-** | NT | **-** | **-** |
| 7 | **-** | **-** | **-** | **-** | **+** | **-** | **-** | NT | **-** | **-** |
| 8 | **-** | **-** | **-** | **-** | **-** | **+** | **-** | NT | **+** | **-** |
| 9 | **-** | **+** | **-** | **-** | **+** | **-** | **-** | NT | **-** | **-** |
| 10 | **+** | **-** | **+** | **-** | **-** | **+** | **-** | NT | **+** | **-** |
| 11 | **+** | **-** | **+** | **-** | **-** | **+** | **-** | NT | **+** | **-** |
| 12 | **+** | **-** | **-** | **-** | **+** | **+** | **-** | NT | **-** | **-** |
| 13 | **+** | **-** | **-** | **-** | **-** | **+** | **-** | NT | **-** | **-** |
| 14 | **+** | **-** | **-** | **-** | **-** | **+** | **-** | NT | **-** | **-** |
| 15 | **+** | **-** | **-** | **-** | **-** | **+** | **-** | NT | **-** | **-** |
| 16 | **+** | **-** | **-** | **-** | **-** | **+** | **+** | NT | **+** | **+** |
| 17 | **+** | **-** | **-** | **+** | **-** | **+** | **+** | NT | **+** | **+** |
| 18 | **-** | **-** | **-** | **-** | **-** | **-** | **+** | NT | **-** | **+** |
| 19 | **+** | **-** | **-** | **-** | **-** | **-** | **+** | NT | **-** | **+** |
| 20 | **+** | **-** | **-** | **-** | **-** | **-** | **+** | NT | **-** | **+** |
| 21 | **-** | **-** | **-** | **-** | **-** | **-** | **+** | NT | **-** | **-** |
| 22 | **+** | **-** | **-** | **-** | **-** | **+** | **+** | NT | **+** | **+** |
| 23 | **+** | **-** | **-** | **-** | **-** | **+** | **-** | NT | **+** | **-** |
| 24 | **-** | **-** | **-** | **-** | **-** | **-** | **+** | NT | **-** | **+** |
| 25 | **+** | **-** | **-** | **-** | **-** | **+** | **-** | NT | **+** | **-** |
