# Supplementary material for: Development of nucleic acid lateral flow immunoassay for molecular detection of Entamoeba moshkovskii and Entamoeba dispar in stool samples
Source: Sci Rep. 2024 Mar 19;14:6635. doi: 10.1038/s41598-024-57332-3 (PMC10951296; doi:10.1038/s41598-024-57332-3)
Supplement: Supplementary file 2 — Supplementary Legends. [file 41598_2024_57332_MOESM2_ESM.docx]

**Figure legend**

**Supplementary Figure 1.** Detection of *E. moshkovskii* PCR products by gel electrophoresis from tested stool samples (a). Lane M is a 100 bp molecular ladder (Thermo Fisher Scientific Inc., USA); lane 1, negative control; lane 2, *E. moshkovskii* positive control; lanes 3-19 are tested stool samples. Lane 13 is *E. moshkovskii* positive sample showing 580 bp PCR product whereas the other samples are negative. Detection of *E. dispar* PCR products by gel electrophoresis from tested stool samples (b). Lane M is a 100 bp molecular ladder; lane 1, negative control; lane 2, *E. dispar* positive control; lanes 3-19 are tested stool samples. Lanes 6 and 15 are *E. dispar* positive samples presenting 752 bp PCR product whereas the other samples are negative.
