# Supplementary figures and images for: Development of nucleic acid lateral flow immunoassay for molecular detection of Entamoeba moshkovskii and Entamoeba dispar in stool samples
Source: Sci Rep. 2024 Mar 19;14:6635. doi: 10.1038/s41598-024-57332-3 (PMC10951296; doi:10.1038/s41598-024-57332-3)

a


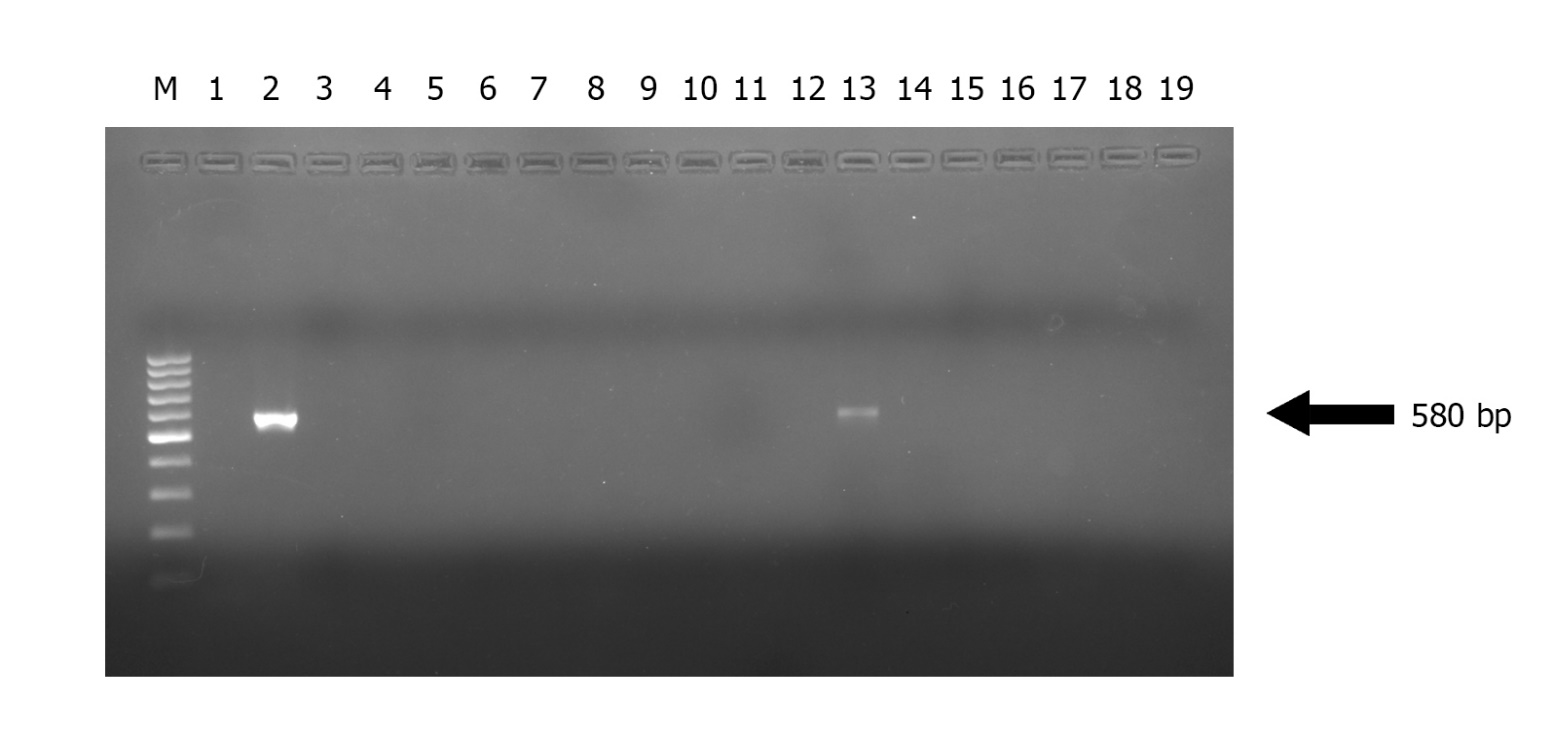


b


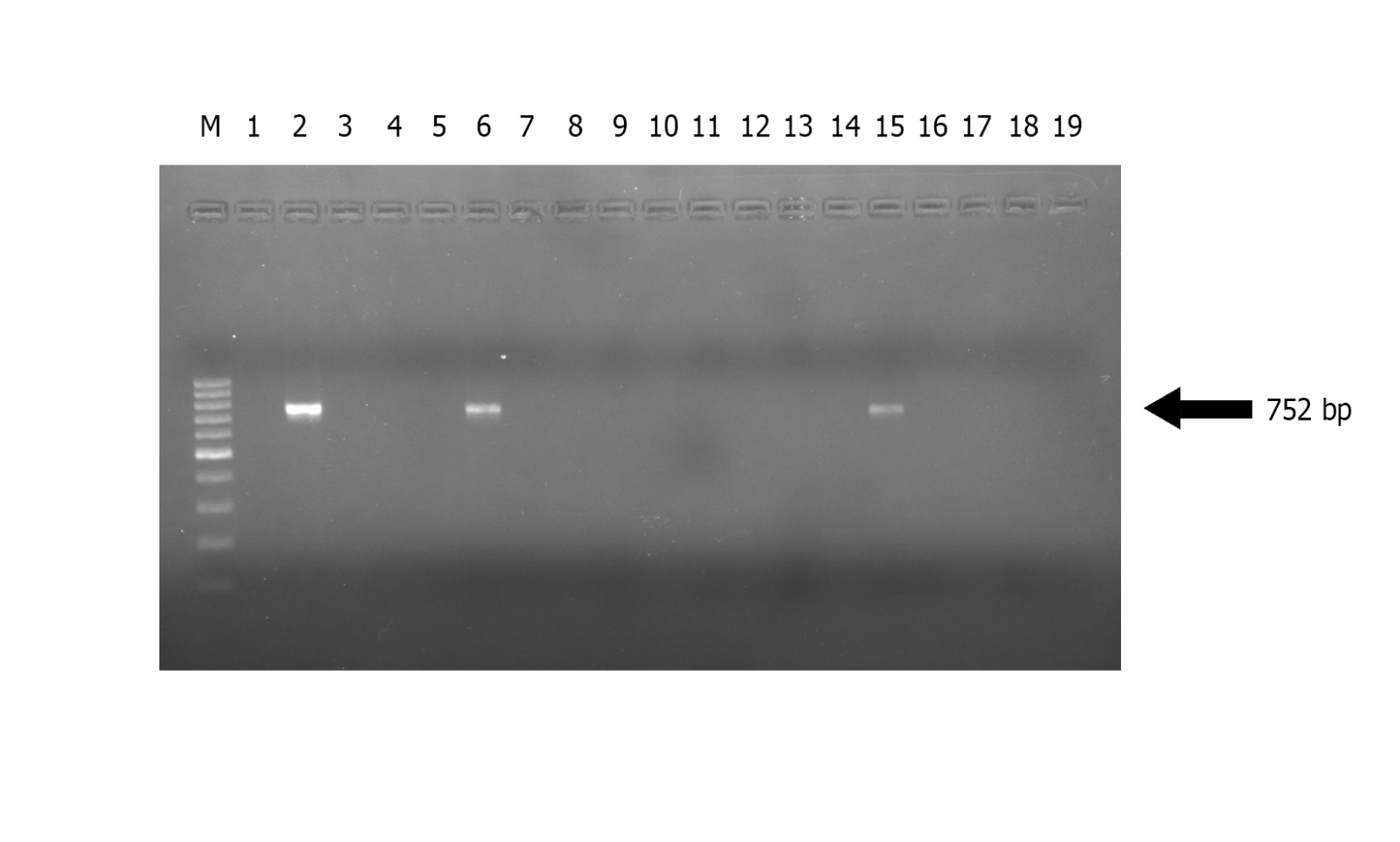

Supplement: Supplementary file 3 — Supplementary Figure 1. [file 41598_2024_57332_MOESM3_ESM.docx]
